# Influence of T Cell Coinhibitory Molecules on CD8^+^ Recall Responses

**DOI:** 10.3389/fimmu.2018.01810

**Published:** 2018-08-08

**Authors:** Anna B. Morris, Layne E. Adams, Mandy L. Ford

**Affiliations:** Department of Surgery, Emory University, Atlanta, GA, United States

**Keywords:** recall, CD8, coinhibitory, memory, cancer, transplant, vaccine, autoimmunity

## Abstract

T cell co-signaling molecules play an important role in fine-tuning the strength of T cell activation during many types of immune responses, including infection, cancer, transplant rejection, and autoimmunity. Over the last few decades, intense research into these cosignaling molecules has provided rich evidence to suggest that cosignaling molecules may be harnessed for the treatment of immune-related diseases. In particular, coinhibitory molecules such as programmed-death 1, 2B4, BTLA, TIGIT, LAG-3, TIM-3, and CTLA-4 inhibit T cell responses by counteracting TCR and costimulatory signals, leading to the inhibition of proliferation and effector function and the downregulation of activation and adhesion molecules at the cell surface. While many reviews have focused on the role of coinhibitory molecules in modifying primary CD8^+^ T cell responses, in this review, we will consider the complex role of coinhibitory molecules in altering CD8^+^ T cell recall potential. As memory CD8^+^ T cell responses are critical for protective memory responses in infection and cancer and contribute to potentially pathogenic memory responses in transplant rejection and autoimmunity, understanding the role of coinhibitory receptor control of memory T cells may illuminate important aspects of therapeutically targeting these pathways.

## Introduction

Recently, there has been an explosion of research on the function of coinhibitory receptors on CD8^+^ T cells, mostly focusing on their role during primary responses [reviewed in Ref. (1–6)]. Here, we will discuss the roles of individual coinhibitory molecules specifically on the recall response of CD8^+^ T cells. Memory cells typically express one or more coinhibitory receptors (7), and as memory cells are important protective regulators against infections and cancer and can be pathogenic in autoimmunity and transplantation, understanding the role of coinhibitory molecules on their recall potential has numerous implications in vaccine design and therapeutics. Two perspectives will be reviewed here: first, that coinhibitory molecules limit recall potential by inhibiting proliferation and activation of secondary effectors, and second, that coinhibitory molecules limit terminal differentiation to preserve recall potential, a process that leads to a stable population of memory T cells that are able to provide a sustained, protective memory response.

## Coinhibitory Molecules Limit Recall Potential

### Infection-Elicited T Cells

Programmed-death 1 (PD-1) is a hallmark coinhibitory receptor that has been implicated in limiting recall potential in models of viral infection. PD-1 belongs to the Ig superfamily, is expressed by activated T and B cells and constitutively expressed by natural killer (NK) cells and macrophages (8–10). PD-1 contains an immunoreceptor tyrosine-based inhibitory motif (ITIM) and an immunoreceptor tyrosine-based switch motif (ITSM) that both contribute to its inhibitory signaling mechanism.

Programmed-death 1 was implicated in limiting CD8^+^ recall responses in studies aiming to understand the high incidence of reinfection of lower respiratory infections in children, which typically indicates poorly generated immunity (11, 12). Interestingly, it was found that dysfunction of pulmonary antigen-specific CD8^+^ T cells generated from influenza and human metapneumonovirus (HMPV) infection in both the primary and secondary effector phase express high levels of PD-1 (13). Upon blockade of PD-1, lytic granule release and antiviral cytokine production were restored, indicating the functional impairment conferred by PD-1 expression. Further, they found that in a model of HMPV reinfection in which B-cell deficient hosts are used to enable reinfection, antigen-specific CD8^+^ T cells further upregulated PD-1, LAG-3, Tim-3, and 2B4 over that of primary effectors (14). Additionally, PD-1 upregulation following primary infection limited recall potential (degranulation and cytokine production) that could be restored with *in vivo* PD-1 blockade at the time of reinfection.

The cosignaling molecule CD244, or 2B4, was also found to play a role in memory CD8^+^ T cell functionality. 2B4, a CD2 family member expressed by NK cells and CD8^+^ T cells, has the unique ability to be costimulatory or coinhibitory due to its ITSM in the cytoplasmic domain (15, 16). Interestingly, microarray data following LCMV Clone 13 infection showed that while some “exhaustive” coinhibitory molecules are similarly expressed in primary and secondary effectors, 2B4 was more highly expressed in the latter (17). Further, studies using antigen-specific CD8^+^ T cells that were genetically deficient in 2B4 revealed that 2B4 expression was associated with lack of survival of secondary effectors in chronic LCMV infection. These data imply that 2B4 limits the recall response of CD8^+^ secondary effector T cells in chronic infection by inhibiting their proliferation and functionality.

Similarly, CTLA-4 blockade during a memory response to *Listeria monocytogenes* enhances CD8^+^ memory T cell recall with greater production of IFNγ and TNF (18). The coinhibitory molecule CTLA-4 outcompetes the costimulatory molecule CD28 for the shared ligands CD80 and CD86 due to its higher affinity (19–21). Importantly, CTLA-4 inhibits T cell activation by numerous mechanisms, including intrinsically *via* interaction with the signaling modalities SHP-2 and PP2A and extrinsically *via* competition for the ligands of CD28 (22–25). Pedicord et al. found that not only does blockade of CTLA-4 during a memory response lead to a better CD8^+^ recall response against bacterial infection but also that anti-CTLA-4 given during the primary response results in an enhanced CD8^+^ memory recall response, suggesting that CTLA-4 upregulation during priming imprints a differentiation program that impedes memory function. These data suggest that PD-1, 2B4, and CTLA-4 all have the ability to inhibit protective memory responses and implicate these inhibitory molecules as potential targets in vaccination strategies to enhance CD8^+^ memory T cell formation and recall potential.

### Vaccination-Elicited T Cells

In the setting of vaccination, inhibitory receptor expression, specifically Tim-3 and PD-1, has been associated with poor protective potential and unsuccessful vaccination strategies. Tim-3, or T cell immunoglobulin mucin-3, is expressed by a myriad of immune cells, including CD8^+^ T cells (26, 27), and its inhibitory function has been identified in models of autoimmunity (26, 28, 29). Vaccination with the Adenovirus5 vector (Ad5), although highly immunogenic, induced higher expression of PD-1 and Tim-3 on memory CD8^+^ T cells and inhibited recall upon boosting compared with alternative Ad vectors (30). Interestingly, when lower doses of Ad5 were administered, the expression of PD-1 and Tim-3 was lowered and overall expansion of CD8^+^ T cells was higher, demonstrating that a more robust CD8^+^ recall potential correlated with lower coinhibitory expression. This also suggests that antigen dose could play a role in the differentiation of CD8^+^ memory T cells that results in upregulation of coinhibitory molecules and inhibition of recall.

Another study corroborated these results using LPG (*Leishmania* lipophosphaglycan) as a vaccine candidate against *Leishmania* infections (31). Vaccination with LPG did not protect mice from *Leishmania mexicana* infection, and LPG vaccination resulted in upregulation of PD-1 on CD8^+^ T cells. They also found, like the study discussed above, that PD-1 upregulation was dose dependent based on the amount of LPG given. They hypothesized that PD-1 could lead to repressed IFNγ production and cytotoxicity, which are important protective modulators in *Leishmania* infections. These studies highlight the role of PD-1 in inhibiting CD8^+^ recall potential, and a possible strategy to avert PD-1 expression with lower antigen doses.

### T Cells in Transplantation and Autoimmunity

Studies in transplantation and autoimmunity have likewise revealed similar associations between coinhibitory molecule expression and CD8^+^ memory recall potential. In both transplantation and autoimmunity, it is beneficial to inhibit allo- or autoreactive CD8^+^ memory T cells to prevent rejection or pathogenic T cell responses, but also to maintain memory CD8^+^ T cell populations to respond to subsequent infections. In work assessing liver transplant patients, it was found that there was an association in the pre-transplant frequency of PD-1 and Tim-3 double-positive CD8^+^ effector memory T cells in patients who would go on to develop liver infections (32), suggesting that these coinhibitory molecules could inhibit the recall potential of these CD8^+^ memory T cells. Furthermore, the frequency of PD-1^+^ and Tim-3^+^ cells also negatively correlated with IFNγ production, indicative of lack of function. Although coinhibitory molecules are typically beneficial in graft survival, this study provided evidence that coinhibitory molecules, especially memory cells expressing both PD-1 and Tim-3, could inhibit the recall potential and memory function of protective antigen-specific cells, leading to increased infections posttransplant.

Additionally, the coinhibitory molecule CTLA-4 has been associated with diminished recall potential of CD8^+^ memory T cells in transplantation and autoimmunity. Studies have used therapeutics to target costimulatory molecules; for instance, CTLA-4Ig, which binds CD80 and CD86 and prevents their binding to CD28 and CTLA-4, and anti-CD28 domain antibodies (anti-CD28 dAb) that specifically block CD28 but preserve CTLA-4-mediated coinhibition (33–35). A recent study by Liu et al. revealed differential outcomes of graft-specific CD8^+^ memory T cells upon treatment with CTLA-4 Ig and anti-CD28 dAb in a murine model of skin transplantation (36). Interestingly, the selective CD28 domain antibodies more potently attenuated graft rejection mediated by memory T cells over CTLA-4 Ig, indicating that CTLA-4 and CD28 are important modulators of memory CD8^+^ T cell recall responses. Although the number of CD8^+^ secondary effectors was similar with either treatment, the cytokine production of the effectors generated with anti-CD28dAb treatment was much lower than that in the CTLA-4 Ig group, signifying a necessary inhibitory role of CTLA-4 in controlling the cytokine production of CD8^+^ secondary effectors. A corroborative study analyzing pathogenic memory CD8^+^ T cells in autoimmunity recapitulated these results (37). Using both human memory CD8^+^ T cells and non-human primate recall studies, they showed that use of a selective CD28 antagonist prevented reactivation and controlled both cellular and humoral memory recall. The results of these studies provide evidence that CTLA-4 has a unique functional role in modulating memory CD8^+^ T cell recall responses.

**Figure 1:**
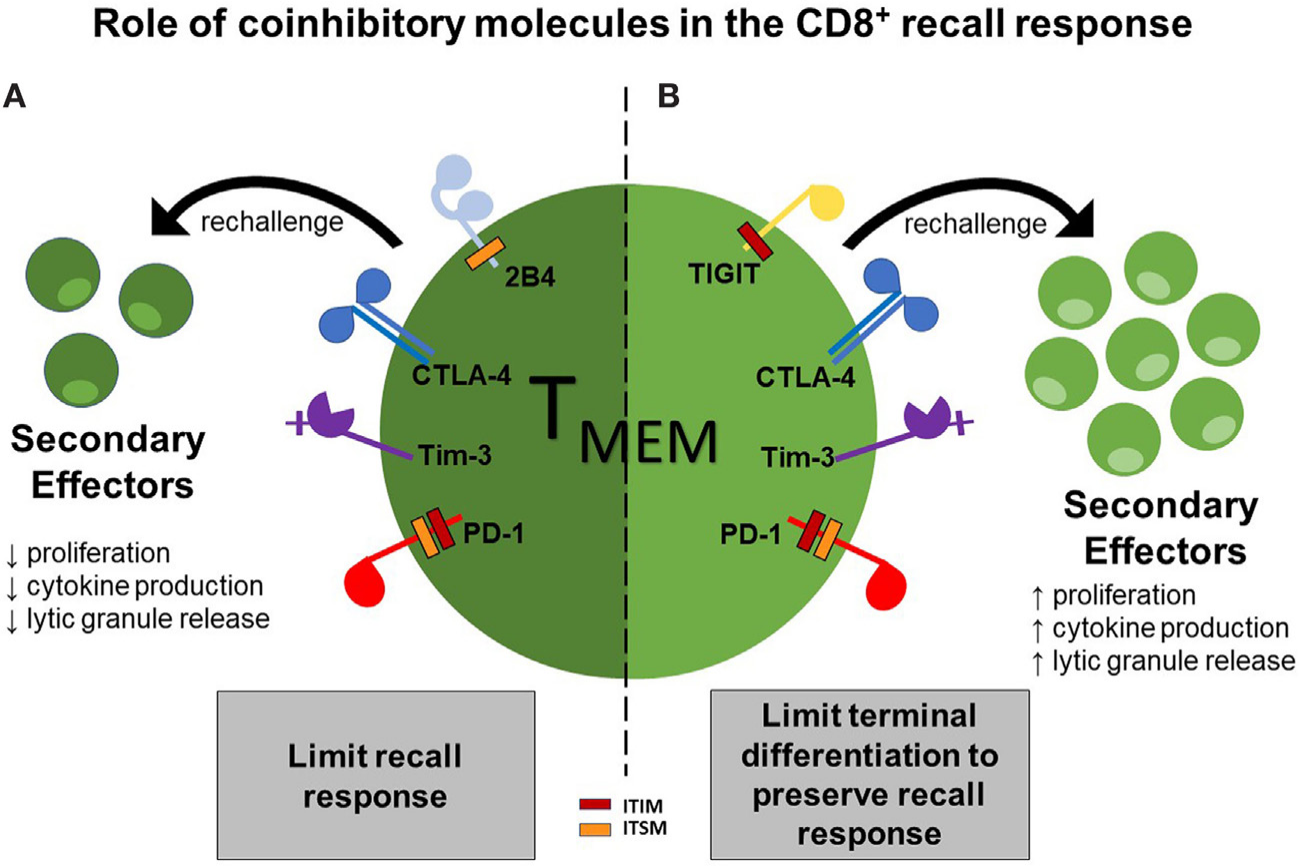
Two functions of coinhibitory molecules in modulating the CD8^+^ recall response. Functionality of CD8^+^ T cell secondary effectors can be limited by ligation of the coinhibitory molecules 2B4, CTLA-4, Tim-3, and programmed-death 1 (PD-1), thus dampening the recall response **(A)**; however, ligation of the coinhibitory molecules TIGIT, Tim-3, CTLA-4, and PD-1 can function to preserve secondary recall responses by inhibiting terminal differentiation, thus leading to a more stable memory population **(B)**.

Moreover, additional studies in a murine transplant model found that 2B4 is also associated with a diminished recall response (Laurie et al., in press). In this model, *L. monocytogenes*-infected animals were rechallenged with a skin graft. Interestingly, the 2B4-deficient CD8^+^ secondary effectors had a significantly higher frequency of IFN-γ and IL-2-secreting cells, as compared to their wild-type counterparts, indicating that 2B4 expressed on CD8^+^ secondary effectors inhibits their ability to secrete cytokines under these conditions. Altogether, these data have provided evidence that coinhibitory molecules, including 2B4, CTLA-4, Tim-3, and PD-1, can selectively limit recall responses *via* inhibition of proliferation, cytokine production, and cytotoxic granule release (Figure 1A).

## Coinhibitory Molecules may Limit Terminal Differentiation to Preserve Recall Potential

Contrary to the concept that coinhibitory receptors have a negative impact on recall potential, recent studies also suggest that coinhibitory receptors may not necessarily negatively impact CD8^+^ memory T cell responses. Although PD-1 is the most well-known exhaustion marker in chronic infection and cancer, and above, we have provided evidence of its ability to inhibit memory CD8^+^ T cell responses, studies revealed that healthy human adults harbor populations of CD8^+^ effector memory T cells that have high levels of PD-1 on their surface, and that these cells were less terminally differentiated (38). Further studies of healthy human CD8^+^ T cells that analyzed multiple inhibitory receptors, including PD-1, CTLA-4, Tim-3, LAG3, 2B4, BTLA, and CD160 found that the expression of inhibitory receptors is not as tightly linked to exhaustion as it is to T cell differentiation or activation status (39), reviewed in Ref. (40). Unlinking coinhibitory molecules and exhaustion status may be an important aspect of understanding the function of these inhibitory molecules on CD8^+^ T cells. Instead of dictating exhaustion status, terminal differentiation, or lack of function, coinhibitory molecules under some conditions limit terminal differentiation and facilitate the CD8^+^ T cell population to be stably maintained.

### Resident Memory T Cells

Interestingly, recent findings suggest that resident memory CD8^+^ T cells (Trm) express coinhibitory molecules in their core gene signature (41, 42). For instance, brain CD8^+^ Trm have not only been shown to express PD-1, but the promotor of *Pdcd1* is epigenetically fixed in a demethylated state, indicating its significance as part of the core gene signature (42). CD8^+^ Trm cells are sentinels for immune surveillance and protection, yet, their secondary effector function has been debated due to their slow turnover and expression of inhibitory molecules, typically indicative of terminal differentiation. In a recent study, it was found that although CD8^+^ Trm maintain high amounts of coinhibitory receptors (i.e., 2B4, CTLA-4, LAG3, PD-1, and Tim-3) relative to circulating memory cells in the spleen, they were still able to undergo local proliferation after secondary rechallenge (43). These findings indicate that the cells expressing these coinhibitory molecules were not terminally differentiated and instead maintained recall potential. Additionally, the findings that coinhibitory molecules are in the gene signature and that the PD-1 promotor is in an epigenetically fixed state indicate a potential function of coinhibitory molecules to modulate CD8^+^ Trm cells in a manner that allows them to be maintained as a stable population capable of recall.

### Decidual T Cells

Coinhibitory molecules have also been associated with a special type of CD8^+^ T cell at the maternal–fetal interface. These decidual CD8^+^ T cells are effector-memory T cells critical to maintain immunity to infection and provide tolerance against the foreign fetus. Recently, it has been shown that these decidual CD8^+^ T cells express little perforin or granzyme B but can respond to viral and bacterial antigens (44–46). Further analysis revealed that although these CD8^+^ effector memory T cells express high levels of PD-1, CTLA-4, TIGIT, and LAG3, they were still able to produce TNF and IFNγ and upregulate perforin and granzyme upon *ex vivo* stimulation. Moreover, although they were slower to begin proliferating, they reached a similar proliferation index as the peripheral CD8^+^ T cells (47). These data indicate that although these memory CD8^+^ T cells express coinhibitory molecules, these receptors do not render them nonfunctional and exhausted. Rather, these CD8^+^ T cells are adequately able to respond to antigen. In the setting of maternal–fetal interface in which tolerance must be maintained to the fetus but protection against infections must also be maintained, the expression of these coinhibitory molecules does not fully render these CD8^+^ T cells exhausted, indicating that expression of coinhibitory molecules could raise the threshold of activation while preventing terminally differentiation or exhaustion.

### Tumor-Infiltrating and Infection-Elicited T Cells

The same phenomenon in which CD8^+^ T cells express coinhibitory molecules that do not necessarily result in exhaustion has been observed in the field of tumor immunology as well. In a study analyzing the molecular signature of tumor-infiltrating lymphocytes (TILs) of non-small cell lung carcinoma, it was found that the CD8^+^ TILs that infiltrated tumors at a high density had high levels of PD-1 and the costimulatory molecule 4-1BB (48), molecules that are upregulated upon TCR engagement and have been associated with both exhaustion and activation (4, 49, 50). These antigen-specific CD8^+^ TILs also exhibited high expression of Tim-3, LAG3, and TIGIT. For instance, it is known that patients with tumors containing a high density of TILs have better survival (48), even if those cells express high levels of coinhibitory molecules. This observation suggests that, in some instances, TILs expressing coinhibitory molecules are still able to elicit antitumor effector function. These data could be reflective of the fact that many exhaustion markers are upregulated as a result of antigen recognition, thus serving as a marker of T cell activation. Alternatively, they could indicate that Tim-3, LAG3, and TIGIT might have additional positive roles on T cell effector function. Support for this hypothesis comes from recent research on Tim-3 in the setting of infection, where a positive impact of Tim-3 on T cell effector function was identified [reviewed in Ref. (51)]. Likewise, in this infection model, expression of Tim-3 on T cells increased signaling downstream of the TCR (52), and Tim-3 deficiency led to impaired CD8^+^ recall responses (53).

Further, in studies assessing the function of CD8^+^ T cells in patients with stage IV advanced metastatic melanoma, TIGIT was found to be co-expressed with PD-1 on tumor-specific effector memory T cells, and TIGIT-expressing cells represented an activated T cell phenotype with high expression of HLA-DR and CD38 (54). The T cell immunoglobulin and ITIM domain (TIGIT) is a member of the Ig superfamily and functions as a coinhibitory molecule on activated T cells, memory T cells, some Tregs, Tfh, and NK cell (55–57). Signaling of TIGIT in T cells leads to the downregulation of the TCR and CD3 molecules and other internal signaling molecules necessary for T cell activation (57). When assessing cytokine production and the ability of these memory T cells to respond to antigen, TIGIT^+^PD-1^+^, TIGIT^-^PD-1^+^, and TIGIT^+^PD-1^−^ all had similar cytokine-producing abilities, whereas Tim3 expression was associated with lower IL-2 and TNF production. These results indicate that TIGIT itself or with PD-1 was not a marker of dysfunction in melanoma, unlike Tim-3. Interestingly, dual blockade of TIGIT and PD-1 led to increased proliferation and cytokine production, indicating both the inhibitory role of TIGIT on CD8^+^ T cells and the elasticity of TIGIT-expressing cells to produce cytokine, indicating a non-terminally differentiated state. Upregulation of TIGIT itself does not lead to decreased cytokine production and recall potential, but could be acting to inhibit T cell activation in a manner that prevents activation-induced cell death while maintaining basal levels of cytokine production.

Consequently, these data have provided evidence that coinhibitory molecules, including TIGIT, Tim-3, CTLA-4, and PD-1, can function to preserve recall response by potentially limiting terminal differentiation, allowing for sufficient cytokine production and cytotoxic granule release (Figure 1B).

## Conclusion

Here, we have discussed the evidence that coinhibitory molecules limit recall potential of CD8^+^ memory T cells by inhibiting expansion and function, but also that coinhibitory molecules can allow for the maintenance of stable memory populations that can respond to rechallenge. Interestingly, certain coinhibitory molecules have been reported to do both, including PD-1 and CTLA-4. The difference in function of the coinhibitory molecule—whether it limits recall potential or maintains stable recall potential—could depend on many factors including the environment in which these cells develop and differentiate, genetic programming imprinted upon priming, duration of antigen exposure, number of coinhibitory molecules, and epigenetic modulations. Understanding the context in which these coinhibitory molecules function is important to instruct better vaccine strategies and immunotherapies for cancer, transplant, and autoimmune diseases.

## Author Contributions

AM and MF conceived of the manuscript focus, researched papers, and wrote the manuscript. LA created the figure. AM, LA, and MF edited the manuscript.

## Conflict of Interest Statement

The authors declare that the research was conducted in the absence of any commercial or financial relationships that could be construed as a potential conflict of interest.
